# MRC2 Promotes Proliferation and Inhibits Apoptosis of Diabetic Nephropathy

**DOI:** 10.1155/2021/6619870

**Published:** 2021-04-28

**Authors:** Lanlan Li, Xin Chen, Henglu Zhang, Min Wang, Weiping Lu

**Affiliations:** Department of Endocrinology and Metabolism, The Affiliated Huai'an No. 1 People's Hospital of Nanjing Medical University, Huai'an, Jiangsu 223300, China

## Abstract

Diabetic nephropathy (DN) is an important microvascular complication of diabetes and is the main cause of end-stage renal disease. Type 2 mannose receptor C (MRC2) is a member of the mannose receptor protein family, which has been confirmed to have the ability to promote the cell migration signaling pathway and invasion. By complementary DNA chip screening and analysis, we found that the expression of MRC2 was upregulated in the kidneys of mice with diabetic nephropathy. However, the role of MRC2 in diabetic nephropathy is still unclear. This work studied the effect of MRC2 on diabetic nephropathy. After verifying the results of the chip by quantitative real-time polymerase chain reaction (qRT-PCR) and western blotting, we used small interfering RNAs (siRNAs) to knock down the expression of MRC2 in mouse mesangial cells (MMCs) and analyzed the level of cell proliferation and apoptosis using western blotting, Cell Counting Kit-8, and flow cytometry. The results showed that the MRC2 knockdown inhibited MMC proliferation and induced cell apoptosis. These results suggest that MRC2 may be a molecular marker and a therapeutic target for diabetic nephropathy.

## 1. Introduction

Diabetic nephropathy (DN) is an important microvascular complication of diabetes and is the main cause of end-stage renal disease. The prevalence of DN in patients with diabetes is 20%–40% [1]. Approximately 30% of patients with type 1 diabetes and 20% of patients with type 2 diabetes develop DN, with end-stage renal disease accounting for approximately 50% of the leading causes of death from chronic kidney disease [2]. Its pathogenesis is complex and is closely related to many factors, such as oxidative stress, cell proliferation, inflammatory reaction, glucose and lipid metabolism disorders, and genetic susceptibility [3, 4]. Mesangial cells are the most affected cells in DN that are trigged to proliferate under high-glucose conditions. At the same time, they secrete a large number of cytokines and synthesize a large amount of mesangial matrix, resulting in glomerular hypertrophy, glomerular basement membrane thickening, and extracellular matrix (ECM) accumulation, eventually leading to glomerulosclerosis [5]. Currently, DN treatment mainly controls hypoglycemia, blood pressure, and lipid regulation, but its therapeutic effects are limited; therefore, exploring the molecular mechanism of glomerular mesangial cell proliferation is important to understand DN and its development, which will facilitate the search for potential therapeutic targets.

The type 2 mannose receptor C (MRC2), also known as uPARAP/Endo180, belongs to the mannose receptor family. Its extracellular domain is divided into three parts, including a cysteine-rich domain, a type II fibronectin (FnII) domain, and eight C-type lectin-like domains from the N-terminal [6, 7]. Studies have shown that the N-terminal cysteine-rich domain of MRC2 may mediate protein-carbohydrate or protein-protein interactions. The FnII domain is usually related to collagen binding. The main roles of MRC2 include the remodeling of the ECM caused by the interaction between its FnII domain and collagen and the ability to promote the cell migration signaling pathway and invasion [8]. Some studies have shown that the turnover rate of the three-dimensional collagen matrix is a key regulator of tumor cell proliferation [9]. MRC2 is the central component of collagen turnover regulated by many mesenchymal cells. Studies have found that the expression of MRC2 is upregulated in gliomas, breast cancer, prostate cancer, and other cancer tissues [10]. However, there are few studies and reports on its regulation in DN. Our group previously studied the MRC2 expression in a DN mouse model using sequencing [11], The results showed that the expression of MRC2 in DN mice was significantly higher than that in normal mice. Therefore, we speculate that MRC2 may affect the occurrence and the development of DN.

## 2. Materials and Methods

### 2.1. Chemicals and Reagents

Fetal bovine serum, Dulbecco's modified Eagle's medium, penicillin-streptomycin solution, and 0.25% trypsin were purchased from Gibco (MA, USA). Lipofectamine™ 2000 and TRIzol were obtained from Invitrogen (MA, USA). HiScript III RT SuperMix for qPCR (+gDNA wiper) was obtained from Vazyme Biotech (Nanjing, China). The antibodies are as follows: anti-GAPDH antibody (1 : 5000, ab8245, Abcam), anti-tubulin antibody (1 : 2000; ab7291, Abcam), anti-cyclin D1 antibody (1 : 2000; 26939-1-AP, Proteintech), anti-proliferating cell nuclear antigen (PCNA) antibody (1 : 1000; ab29, Abcam), and anti-MRC2 antibody (1 : 2000; PA5-50956, Thermo Fisher). Horseradish peroxidase-conjugated anti-rabbit immunoglobulin G (IgG; 1 : 10000, Proteintech) and anti-mouse IgG (1 : 10000, Proteintech) were used as secondary antibodies. A protein extraction kit was from Beyotime Biotechnology (Beijing, China). Cell Counting Kit-8 was purchased from Dojindo (Tokyo, Japan).

### 2.2. Patients and Clinical Specimens

The patients who were treated at the Huai'an First People's Hospital affiliated to Nanjing Medical University from November 2019 to September 2020 were divided into two groups. The first group comprised 21 patients with DN (13 males and 8 females, mean age 55.1 ± 7.4 years old). In addition, 21 healthy subjects who underwent physical examination at the same time were selected as the healthy control group (12 males and 9 females, mean age 55.5 ± 7.2 years old). Remaining peripheral blood samples from the two groups were collected. According to the instructions, total RNA was extracted from the samples using TRIzol (Invitrogen, MA, USA), and cDNA was synthesized using HiScript III RT SuperMix for qPCR (Vazyme Biotech, Nanjing, China) and stored at -80°C. The study was approved by the hospital ethics committee (ethical approval number YX-2020-004-01.)

### 2.3. Animal Model

Eight-week-old specific pathogen-free, obese, type 2 diabetic animal model BKS-Leprem2Cd479/Gpt mice and BKS-Leprem2Cd479/Gpt control mice were purchased from the Model Animal Research Center of Nanjing University (Nanjing, China). The mice were fed for one week to allow adaptation, and parameters such as blood glucose, body weight, urine volume, and 24 h urinary albumin were measured. The kidney tissues of mice were removed and stained with hematoxylin and eosin (H&E) to confirm the diagnosis of DN. The program was approved by the Animal Care and Ethics Committee of Huai'an First People's Hospital, Nanjing Medical University (ethical approval number DW-P-2020-002-01), and all procedures adhered to animal care guidelines.

### 2.4. Cell Culture and Transfection

Mouse mesangial cells (MMCs) were purchased from the Shanghai Cell Bank of the Chinese Academy of Sciences. To simulate nondiabetic and diabetic conditions, MMCs were cultured in Dulbecco's modified Eagle's medium (DMEM)/F-12 (3 : 1) complete medium with a glucose concentration of 5 mmol/L (low-glucose group) and 25 mmol/L (high-glucose group) containing 5% fetal bovine serum, 100 U/mL penicillin, and 100 *μ*g/mL streptomycin [12]. The experiment was carried out with 3-8 passages of well-growing MMCs.

Small interfering RNAs (siRNAs) against MRC2 (si-MRC2 1 and si-MRC2 2) and negative control (si-NC) were designed and synthesized by RiboBio (Guangzhou, China). MMCs were inoculated into a 6-well culture plate at a density of 1 × 10^5^ cells/well. According to the manufacturer's instructions, siRNAs and si-NCs were transfected into cells with Lipofectamine 2000 (Invitrogen) in serum-free medium and incubated for 6 h; the transfection was then terminated by changing the complete medium. After 48 h of culture, the cells were collected for further analysis.

### 2.5. HE Staining and Immunohistochemistry

The kidney tissues from the mouse control and DN groups were fixed with 4% paraformaldehyde and embedded in paraffin. The wax blocks were sliced into thin slices, each being 5–8 *μ*m thick. The slices were dewaxed in xylene and ethanol and then stained with H&E. For immunohistochemistry, the cut slices were dewaxed with xylene and ethanol, washed with clean water for a period of time, and then blocked with antigen, sealed with serum, and soaked in a primary antibody and secondary antibody and finally in the chromogenic agent. Subsequently, the slides were rinsed with clean water, soaked in hematoxylin for dyeing, cleaned, and dried. The prepared slides were then observed and analyzed using a microscope.

### 2.6. Immunofluorescence Assay

MMCs were inoculated into a 24-well culture plate at a density of 5 × 10^4^ cells/well. MMCs were fixed with 4% paraformaldehyde, treated with 0.1% Triton X-100, and blocked in 5% goat serum solution. Then, the cells were incubated with the primary antibody against MRC2 overnight at 4°C and then incubated with the secondary antibody labeled with cy3 at room temperature for 1 h. The films were sealed after staining with DAPI (4′,6-diamidino-2-phenylindole).

### 2.7. RNA Isolation and Quantitative Real-Time PCR

MMCs were inoculated into a 6-well culture plate at a density of 1 × 10^5^ cells/well. According to the manufacturer's instructions, siRNAs and si-NCs were transfected into cells. Total RNA was extracted from the MMC cell lines, clinical blood samples, and mouse kidney tissues using the TRIzol reagent (Invitrogen) according to the manufacturer' s instructions. The RNA obtained was used to synthesize complementary DNA (cDNA) with the HiScript III RT SuperMix for qPCR (+gDNA wiper) (Vazyme Biotech, Nanjing, China). The ChamQ SYBR qPCR Master Mix (without ROX; Vazyme Biotech) was used to perform quantitative real-time polymerase chain reaction (qRT-PCR) using a Roche LightCycler 480 (Roche, Switzerland). The primers used are as follows (h indicates human, and m indicates mouse):

GAPDH-F(h): 5′-AAGACGGGCGGAGAGAAACC-3′

GAPDH-R(h): 5′-CGTTGACTCCGACCTTCACC-3′

MRC2-F(h): 5′-CCGAAACCGGCTATTCAACCT-3′

MRC2-R(h): 5′-CGGTCACACTCATACATGCCC-3′

*β*-Actin-F(m): 5′-GTGACGTTGACATCCGTAAAGA-3′

*β*-Actin-R(m): 5′-GCCGGACTCATCGTACTCC-3′

MRC2-F(m): 5′-TCTCCCGGAACCGACTCTTC-3′

MRC2-R(m): 5′-AACTGGTCCCCTAGTGTACGA-3′

### 2.8. Cell Proliferation Assay

MMCs with different treatments were seeded in 96-well plates at a density of 2000/well, and three replicate wells were used for each group. The cells were cultured in an incubator (5% CO_2_ at 37°C) for 0, 24, 48, 72, and 96 h, and cell proliferation was measured using Cell Counting Kit-8 (CCK-8; Dojindo Molecular Technologies, Tokyo, Japan), according to the manufacturer's instructions.

### 2.9. Cell Cycle Experiment

The treated cells were inoculated into six-well plates at a density of 1 × 10^6^ cells/well. After 24 h of culture, the cell suspension was collected, fixed with 75% ethanol, and stored in a flow tube at -20°C. The ethanol-fixed cell suspension was then centrifuged at 350g for 5 min. The supernatant was removed, and the pellet was washed thrice with phosphate-buffered saline. The cells were then stained using propidium iodide. A portion (0.5 mL) of the stained cell solution was kept away from light for 1 h at room temperature and was then filtered using a 300 *μ*m mesh filter membrane to remove the cell mass. Each experiment was repeated thrice. The fluorescence data from 20,000 cells were collected and analyzed using FlowJo V10.

### 2.10. Protein Extraction and Western Blot Analysis

MMCs with different treatments were seeded in 6-well plates at a density of 5 × 10^5^/well. Total protein from mouse kidney tissues and MMCs was extracted using ice-cold radioimmunoprecipitation assay (RIPA) lysis buffer containing protease and phosphatase inhibitors (Beyotime Biotechnology, Shanghai, China) in strict accordance with the manufacturer's protocol. Equal amounts of protein per lane (16–20 mg) were loaded onto 10% sodium dodecyl sulfate polyacrylamide gel electrophoresis according to different molecular weights and transferred to polyvinylidene fluoride membranes. The antibodies are as follows: anti-GAPDH antibody (1 : 5000, ab8245, Abcam), anti-tubulin antibody (1 : 2000; ab7291, Abcam), anti-cyclin D1 antibody (1 : 2000; 26939-1-AP, Proteintech), anti-proliferating cell nuclear antigen (PCNA) antibody (1 : 1000; ab29, Abcam), and anti-MRC2 antibody (1 : 2000; PA5-50956, Thermo Fisher). Horseradish peroxidase-conjugated anti-rabbit immunoglobulin G (IgG; 1 : 10000, Proteintech) and anti-mouse IgG (1 : 10000, Proteintech) were used as secondary antibodies. The sizes of the protein bands were quantified using the ImageJ software.

### 2.11. Cell Apoptosis Assay

MMCs with different treatments were seeded in 6-well plates at a density of 5 × 10^5^/well. The apoptosis assay was performed using FITC Annexin V Apoptosis Detection Kit (BD Biosciences, CA, USA). The percentage of apoptotic cells at each stage was detected using FACSCalibur (BD Biosciences).

### 2.12. Statistical Analysis

SPSS 22.0 software was used for the statistical analysis of experimental data, which were presented as means ± standard deviation. Student's *t*-test was applied to analyze experiments involving two groups, while one-way analysis of variance was applied for experiments with no less than three groups. *P* < 0.05 was considered statistically significant.

## 3. Results

### 3.1. MRC2 Was Highly Expressed in Peripheral Blood Samples of DN Patients with DN

**Figure 1:**
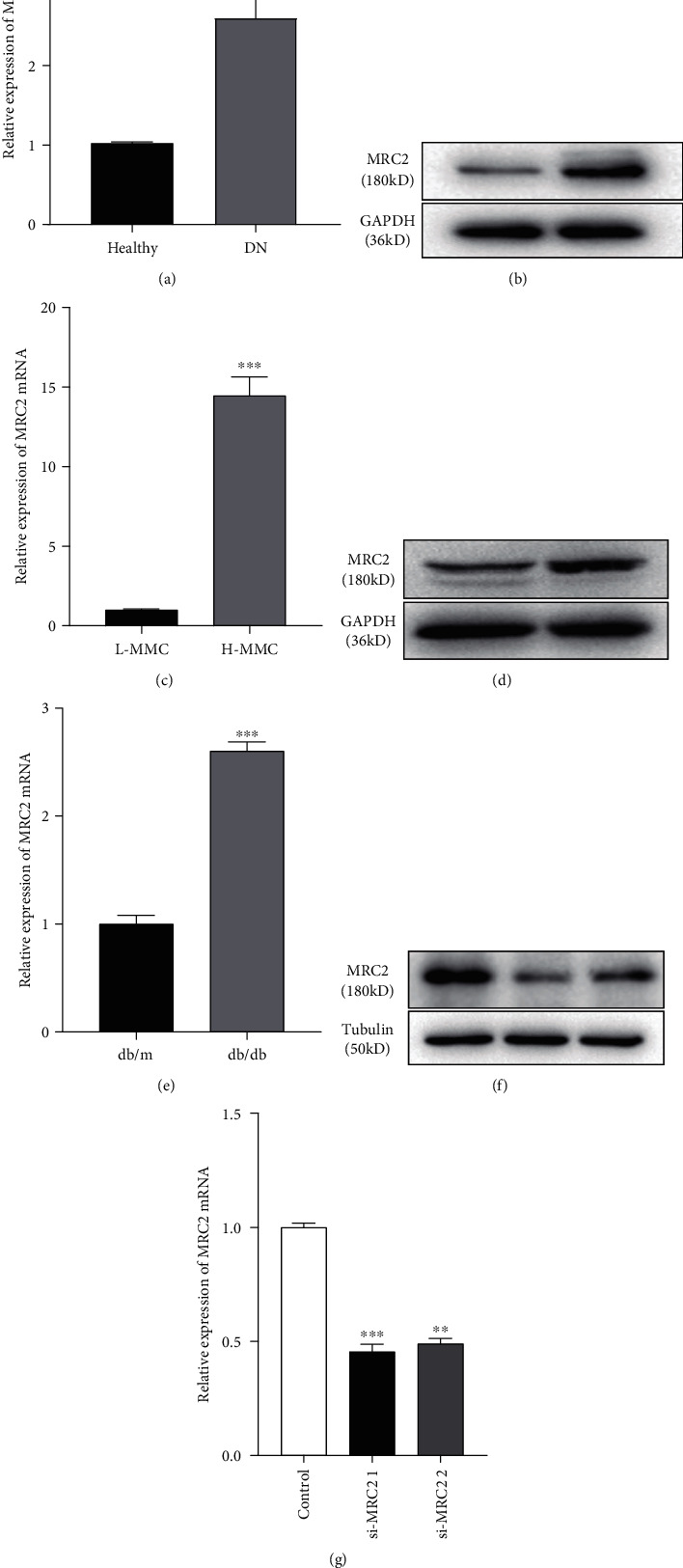
The expression of MRC2 measured in the peripheral blood of 21 healthy subjects and 21 patients with DN detected by qRT-PCR increased in patients with DN (a). MRC2 expression in cell and animal models of DN and MRC2 silencing. The MRC2 expression in MMCs cultured under low-glucose and high-glucose conditions was detected using western blotting (b) and qRT-PCR (c). The MRC2 expression in the renal tissues of normal and DN mice was detected using western blotting (d) and qRT-PCR (e). The MRC2 expression in MMCs transfected with the negative control (si-NC) and siRNAs (si-MRC2 1 and si-MRC2 2) was detected using western blotting (f) and qRT-PCR (g). ^∗∗∗^*P* < 0.001 vs. healthy group. ^∗∗^*P* < 0.01, ^∗∗∗^*P* < 0.001 vs. control group.

To study the difference in MRC2 expression in patients with and without DN, we carried out qRT-PCR assays on peripheral blood samples. The results showed that the expression of MRC2 in the peripheral blood of patients with DN was significantly higher than that in healthy subjects (*P* < 0.05; Figure 1(a)).

### 3.2. MRC2 Was Overexpressed in the DN Mouse Model

**Figure 2:**
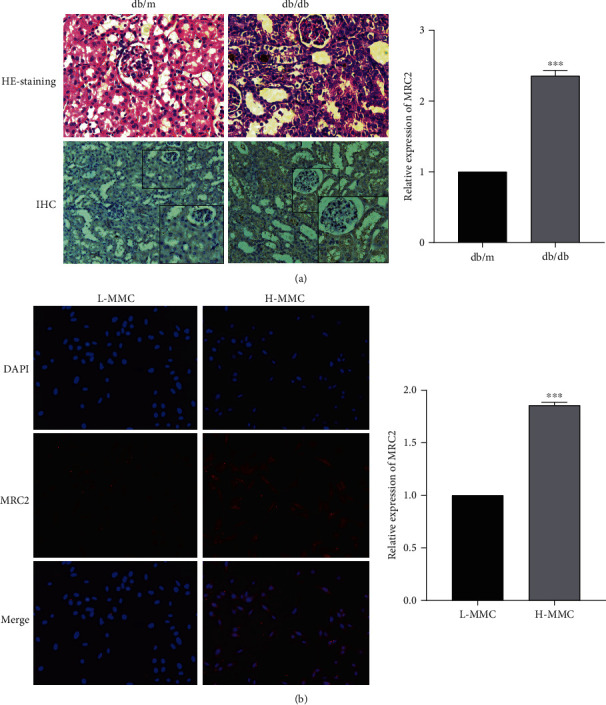
The renal tissues of normal and DN mice were collected for H&E staining and immunohistochemistry analysis (a), and immunofluorescence was used to detect the expression of MRC2 in MMCs cultured under low-glucose and high-glucose conditions (b). ^∗∗∗^*P* < 0.001 vs. control group.

To explore the expression of MRC2 in the kidneys of DN mice, we performed immunohistochemical staining. The results showed that the expression of MRC2 in the kidney of DN mice was significantly higher than that in the control group (*P* < 0.001; Figure 2(a)). Western blotting and qRT-PCR experiments were used to detect the level of MRC2 in normal mice and DN models. The results showed that the expression of MRC2 in the renal tissue of DN mice was significantly higher than that in the control group (*P* < 0.001; Figures 1(d) and 1(e)).

### 3.3. MRC2 Expression Was Upregulated in High Glucose-Induced MMCs

MMCs were cultured in low glucose (5.5 mmol/L) and high glucose (25 mmol/L) to simulate normal and diabetic physiological environments. The qRT-PCR assay results showed that the expression of MRC2 was significantly increased in cells cultured with high glucose. In addition, we found the same results via western blotting of extracted proteins from cells exposed to low and high glucose (*P* < 0.001; Figures 1(b) and 1(c)). Similarly, the above results were also consistent with our immunofluorescence experiments (*P* < 0.001; Figure 2(b)).

### 3.4. MRC2 Participated in the Proliferation of MMCs

**Figure 3:**
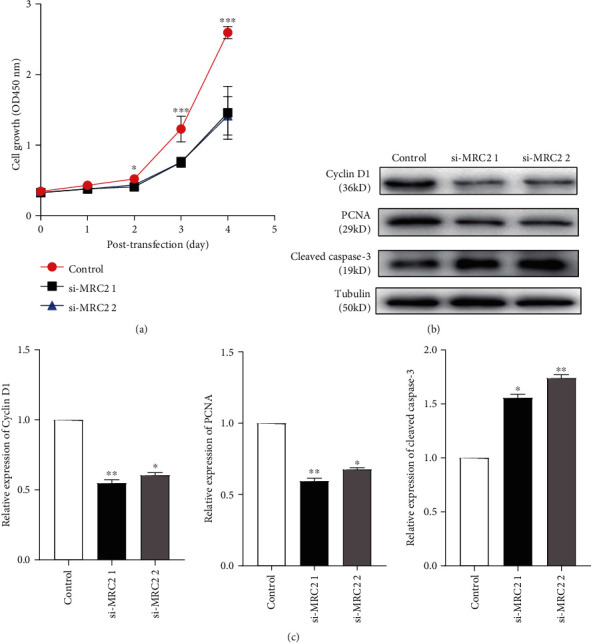
Effect of MRC2 on the proliferation of MMCs. CCK-8 assays were used to detect the proliferation of MMCs transfected with negative control (si-NC) and siRNAs (si-MRC2 1 and si-MRC2 2) for 0, 1, 2, 3, and 4 days under high-glucose conditions (a). The expression of PCNA, cyclin D1, and cleaved caspase-3 in MMCs transfected with negative control (si-NC) and siRNAs (si-MRC2 1 and si-MRC2 2) was detected using western blotting (b). ^∗^*P* < 0.05, ^∗∗^*P* < 0.01, and ^∗∗∗^*P* < 0.001 vs. control group.

We performed the CCK-8 assay to explore the role of MRC2 in cell proliferation. The results showed that the cell proliferation rate of the MRC2 knockdown group was lower than that of the control group (*P* < 0.05; *P* < 0.001; Figure 3(a)). In addition, we found that the MMC expression of PCNA and cyclin D1, which are indicators of proliferation, decreased after knockdown of MRC2 using western blotting (*P* < 0.05; *P* < 0.01; Figures 3(b) and 3(c)). The above data indicate that MRC2 promotes cell proliferation, which may promote the development of DN.

### 3.5. MRC2 Changed the Cell Cycle Progression of MMCs

**Figure 4:**
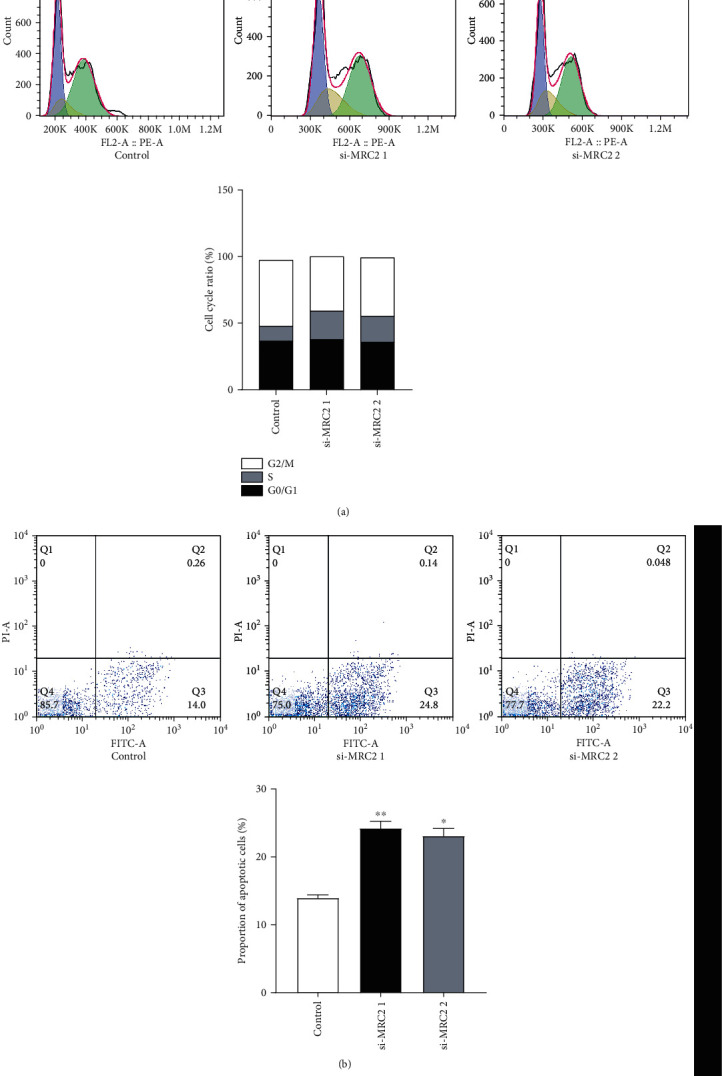
The cell cycle was analyzed using flow cytometry. Compared with the negative control group, the proportion of cells in the S phase in the experimental group increased, the ratio of the cells in the G0/G1 phase remained unchanged, and the ratio of cells in the G2/M phase decreased (a). The number of apoptotic cells was analyzed using flow cytometry. The proportion of apoptotic cells increased after knockdown of MRC2. The experiment was repeated three times (b). ^∗^*P* < 0.05 vs. control group.

Analysis of cell cycle progression in the control and MRC2 knockdown groups revealed that the proportion of cells in the S phase (DNA replication phase) in the experimental group significantly increased compared to the control group (*P* < 0.05), the proportion of cells in the G0/G1 phase (the gap between the completion of mitosis and DNA replication) was unchanged (*P* > 0.05), and the proportion of cells in the G2/M phase was lower than that in the control group (*P* < 0.05; Figure 4(a)). This suggests that the cell cycle progression was delayed after knockdown of MRC2.

### 3.6. MRC2 Inhibited the Apoptosis of MMCs

The results of flow cytometry showed that the number of apoptotic cells in the MRC2 knockdown group was significantly higher than that in the control group (*P* < 0.05; *P* < 0.01; Figure 4(b)). Consequently, western blotting was used to detect the protein expression level of the apoptosis marker caspase-3. The results showed that the concentration of cleaved caspase-3 was significantly higher than that in the control group after knockdown of MRC2, suggesting that MRC2 may play a role in inhibiting cell apoptosis.

## 4. Discussion

As DN is a common clinical complication of diabetes, early diagnosis and treatment of DN are crucial. At present, the most commonly used biomarker for the detection of DN is albuminuria. However, studies have shown that microalbuminuria cannot accurately reflect the occurrence of DN [13]; therefore, it is of great significance to explore further molecular markers and therapeutic targets of DN.

MRC2, a member of the mannose receptor protein family, is mainly expressed by mesenchymal cells such as fibroblasts and osteoblasts and is present in sites that show active tissue remodeling [10]. MRC2 can bind and internalize intact and degraded collagen and then participate in the renewal of collagen in the cell membrane and ECM. Its main function is to promote the initial adhesion of fibroblasts to collagen and accelerate the migration of fibroblasts on the procollagen fiber matrix, thus promoting cell diffusion [14]. Gai et al. [15] have found that MRC2 expression is significantly higher in hepatocellular carcinoma than in paracancerous tissues. It promotes the migration and invasion of hepatocellular carcinoma cells by mediating the degradation of intracellular collagen, which can be used as a prognostic marker for patients with hepatocellular carcinoma. Dong et al. [16] found that the expression of MRC2 increased in developing bones and that collagen decomposition is one of the rate-limiting steps in the process of growth. Studies have found that the expression of MRC2 is upregulated in cancers such as breast and prostate cancers and in diseases such as osteoarthritis and rheumatoid arthritis [7, 17]. However, there are few studies on MRC2 in DN. The previous sequencing results of our group showed that the MRC2 expression in the renal tissues of DN mouse models was significantly higher than that in normal tissues [18]. Therefore, we speculate that this may be related to the occurrence and development of DN.

In this study, we found that the expression level of MRC2 in the peripheral blood of patients with DN was higher than that in healthy subjects. We cultured MMCs with high and low glucose to simulate the physiological environment of normal and diabetic conditions. qRT-PCR and western blotting analysis revealed that the expression of MRC2 was upregulated in cells exposed to high glucose. In a similar manner, we detected its expression in the renal tissues of control and DN mice and found that its expression was also increased in the renal tissues of DN mice. We conducted immunohistochemical and cellular immunofluorescence experiments to further verify these results. Our results were consistent with the sequencing and aforementioned experimental results.

To further study the function of MRC2, we constructed two types of siRNAs to knock down the expression of MRC2 in MMCs and to explore the effect of MRC2 on cell proliferation and apoptosis in DN. The CCK-8 results showed that the growth rate of MMCs with MRC2 knockdown was slower than that of the control group. Furthermore, western blotting analysis showed that the protein expression of cyclin D1 and PCNA, the markers of cell proliferation, decreased, and the expression of cleaved caspase increased after knockdown of MRC2. Similarly, we used flow cytometry to detect the effect of MRC2 on apoptosis and cell cycle of MMCs. The results showed that after knockdown of MRC2, the proportion of apoptotic cells increased, the proportion of cells in the G0/G1 phase remained unchanged, the proportion of cells in the S phase increased, and the proportion of cells in the G2/M phase decreased, resulting in cell arrest.

## 5. Conclusions

In conclusion, our study shows that the expression of MRC2 is increased in the peripheral blood of patients with DN, MMCs cultured with high glucose, and the kidney of DN mice. MRC2 gene knockout can inhibit the proliferation of MMCs and induce apoptosis. We conducted a preliminary study on MRC2 in diabetic nephropathy, which may provide a new molecular biomarker and therapeutic target for the occurrence and development of DN.

## Data Availability

The data used to support the findings of this study are included within the article.

## Abbreviations

MRC2: Type 2 mannose receptor C
DN: Diabetic nephropathy
MMCs: Mouse mesangial cells
DMEM: Dulbecco's modified Eagle's medium
qRT-PCR: Quantitative real-time polymerase chain reaction
PCNA: Proliferating cell nuclear antigen
RIPA: Radioimmunoprecipitation assay
CCK-8: Cell Counting Kit-8
H&E: Hematoxylin and eosin.
